# Deciphering the enzymatic target of a new family of antischistosomal agents bearing a quinazoline scaffold using complementary computational tools

**DOI:** 10.1080/14756366.2020.1712595

**Published:** 2020-01-15

**Authors:** Victor Sebastian-Perez, Alfonso García-Rubia, Sayed H. Seif el-Din, Abdel-Nasser A. Sabra, Naglaa M. El-Lakkany, Samia William, Tom L. Blundell, Louis Maes, Ana Martinez, Nuria E. Campillo, Sanaa S. Botros, Carmen Gil

**Affiliations:** a Centro de Investigaciones Biológicas (CIB-CSIC), Madrid, Spain; b Pharmacology Department, Theodor Bilharz Research Institute, Giza, Egypt; c Parasitology Department, Theodor Bilharz Research Institute, Giza, Egypt; d Department of Biochemistry, University of Cambridge, Cambridge, UK; e Laboratory for Microbiology, Parasitology and Hygiene (LMPH), University of Antwerp, Antwerp, Belgium

**Keywords:** Drug discovery, quinazoline, *Schistosoma mansoni*, target deconvolution

## Abstract

A previous phenotypic screening campaign led to the identification of a quinazoline derivative with promising *in vitro* activity against *Schistosoma mansoni*. Follow-up studies of the antischistosomal potential of this candidate are presented here. The *in vivo* studies in a *S. mansoni* mouse model show a significant reduction of total worms and a complete disappearance of immature eggs when administered concomitantly with praziquantel in comparison with the administration of praziquantel alone. This fact is of utmost importance because eggs are responsible for the pathology and transmission of the disease. Subsequently, the chemical optimisation of the structure in order to improve the metabolic stability of the parent compound was carried out leading to derivatives with improved drug-like properties. Additionally, the putative target of this new class of antischistosomal compounds was envisaged by using computational tools and the binding mode to the target enzyme, aldose reductase, was proposed.

## Graphical Abstract

**Figure:**
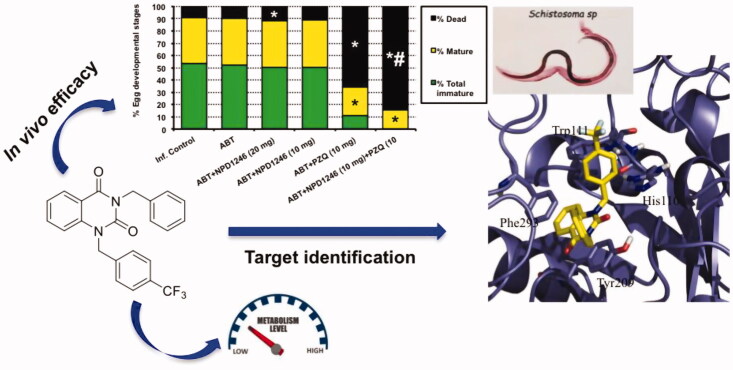

## Introduction

Schistosomiasis is a parasitic infectious disease caused by a trematode belonging to *Schistosoma spp*. Transmission occurs through contact with freshwater that is contaminated with larval forms (furcocercariae). Once in the human body, the larvae become adults in the blood vessels where the females release eggs. Part of the eggs is passed in the faeces or urine to continue the parasite’s life cycle by contaminating water while most become trapped in body tissues causing immune-inflammatory responses and progressive damage to organs^1^. This neglected tropical disease is endemic in a number of tropical and subtropical countries representing a serious health problem especially in poor communities. The disease has recently also reached Europe, demonstrating the possibility to emerge in new geographical areas previously unknown related to migration movements and parasite genetic variants^2^.

Treatment and control of all forms of schistosomiasis fully rely on mass drug administration with the only available antischistosomal drug praziquantel (PZQ)^3^. Even considering it is safe, effective, operationally convenient and low-cost, there is an increasing concern among the scientific community to anticipate PZQ therapeutic failure^4^. The massive use for many years has clearly increased the risk of resistance development. This fact, together with the lack of efficacy against immatures makes the development of new drugs more urgent^5^.

Historically, drug discovery for schistosomiasis has been based on phenotypic screening using whole-organism assays, however, new chemotherapeutics with known mechanism-of-action (MOA) are highly desirable to anticipate drug resistance^6^^,^^7^. Particular advantages and disadvantages of phenotypic vs. target-based approaches are well known, and the combination of both strategies is logically the best way to move forward and optimise the drug discovery process^8^^,^^9^. Recognising the pivotal role of target identification and the challenging task of identifying the MOA for bioactive small molecules, significant progress has been made to develop a number of computational strategies to unveil the MOA of phenotypic hits^10^. *In silico* target identification offers chances for drug repurposing and for the detection of new links between disease and known targets. Large datasets such as ChEMBL^11^ or PubChem^12^ are now available and contain an impressive amount of biological data related to the activity of millions of ligands in multiple assays. As such, they provide an invaluable source of information for the development of knowledge-based approaches guided by computational techniques^13^.

**Figure 1.**
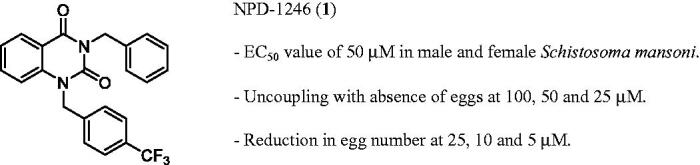
*In vitro* findings for NPD-1246 (**1**) previously reported^14^.

In a previous *in vitro* phenotypic screening campaign using *Schistosoma* *mansoni* with worm killing as primary outcome, compounds from a selected library were successfully classified and prioritised based on potency and selectivity^14^. The present work expands the evaluation of the antischistosomal activity for the quinazoline NPD-1246 (**1**) (Figure 1) which was one of the best *in vitro* “hits”, involving (i) *in vivo* evaluation in the *S. mansoni*-infected mouse model, (ii) a medicinal chemistry programme to obtain better drug-like compounds and (iii) exploration of the putative target using computational consensus methodology applying ligand-based approaches.

## Materials and methods

### Chemical procedures

Substrates were purchased from commercial sources and used without further purification. Melting points were determined with a Mettler Toledo MP70 apparatus. Flash column chromatography was carried out with automated silica gel column chromatography at medium pressure using silica gel (E. Merck, Grade 60, particle size 0.040–0.063 mm, 230–240 mesh ASTM) with the indicated solvent as eluent. Compounds were detected with UV light (254 nm). ^1^H NMR or ^13^C NMR experiments were obtained on the Bruker AVANCE-300 or Bruker AVANCE-500 spectrometers. ^1^H and ^13^C spectra were calibrated using residual chloroform or DMSO as an internal reference (CHCl_3_: δ 7.26 ppm and δ 77.3 ppm, respectively. DMSO: 2.54 ppm and 40.4 ppm, respectively). Chemical shifts for ^1^H NMR are reported as follows: chemical shift (δ ppm), multiplicity (s = singlet, d = doublet, t = triplet, q = quartet, dd = double of doublets, ddd = doublet of double of doublets, td = triplet of doublets, m = multiplet), coupling constant (Hz), and integration. Chemical shifts for ^13^C NMR are reported in terms of chemical shift (δ ppm). Purity of assayed compounds was determined by elemental analysis recorded on Heraeus CHN-O-rapid analyser performed by the analytical department at CAI (UCM) and values were within ±0.4% of the theoretical values for all compounds.

**Method A (R^3^=Me).** General procedure for the synthesis of substituted 3-benzylquinazolin-2,4(1*H*,3*H*)-dione using anthranilate methyl ester derivatives as starting material. In a round‐bottomed flask with a magnetic stir bar, the corresponding anthranilate methyl ester is dissolved in toluene (5 mL/mmol) and cold at 0 °C. 1.1 equiv. of the corresponding isocyanate is added to the mixture, which is stirred overnight at rt. Then, 8 mL of toluene and 2 mL/mmol of NaOH (6 M) is added and the mixture is heated to 70 °C until complete conversion to the 3-substituted quinazolinedione (6 h). The reaction mixture is cooled in an ice bath and water (5 mL/mmol) is added, which causes a precipitate. This slurry is stirred for 30 min, filtered, washed with water (5 mL/mmol), and then heptanes (3 × 10 mL/mmol) before drying the solids under a stream of nitrogen. Products are generally obtained as white or off‐white solids and were used in the next step without further purification.

**Method B (R^3^=H).** General procedure for the synthesis of substituted 3-benzylquinazolin-2,4(1*H*,3*H*)-dione using anthranilic acid derivatives as starting material. In a round‐bottomed flask with a magnetic stir bar, the corresponding anthranilic acid derivative is dissolved in diethyl ether (8 mL/mmol) and 1.1 equiv. of the corresponding isocyanate is added drop by drop to the mixture, which is stirred overnight at rt. The solvent is rotary evaporated and replaced with EtOH (10 mL/mmol). Then, 2 mL/mmol of concentrated HCl (12.1 M) is added and the mixture is heated to 70 °C until complete conversion to the 3‐substituted quinazolinedione (3 h). The reaction mixture is cooled in an ice bath and water (5 mL/mmol) is added, which causes a precipitate. This slurry is stirred for 30 min, filtered, washed with water (5 mL/mmol), and then heptanes (3 × 10 mL/mmol) before drying the solids under a stream of nitrogen. Products are generally obtained as white or off‐white solids and were used in the next step without further purification.

**General procedure for the synthesis of substituted 3-benzyl-1-(4-(trifluoromethyl)benzyl)quinazolin-2,4(1*H*,3*H*)-dione.** In a round‐bottomed flask with a magnetic stir bar, substituted 3-benzylquinazolinedione previously obtained by method A or B is dissolved in DMF (3mL/mmol) and 1.5 equiv. of 4-(trifluoromethyl)benzyl chloride and 3 equiv. of NaHCO_3_ are added. The mixture is heated to 130 °C overnight. The reaction mixture is cooled and water (5mL/mmol) and EtOAc (15 mL/mmol) are added. Upon separation of the layers, the aqueous phase was extracted with EtOAc (3 × 10 mL). The organic layer was washed sequentially with sat. aq. NH_4_Cl, brine, then dried over Na_2_SO_4_ anhydrous. The desiccant was filtered and solvent removed under vacuum. The residue was purified by flash column chromatography using as eluents mixtures of solvents (EtOAc/hexane, 1:9–6:4) as indicated in each case to obtain the desired products.

***3-Benzyl-1-(4-(trifluoromethyl)benzyl)quinazoline-2,4(1H,3H)-dione* (1).** Reagents: 3-benzylquinazolin-2,4(*1H,3H*)-dione (1.2 mmol) obtained by method A from methyl 2-aminobenzoate and 1-(isocyanatomethyl)benzene), 4-(trifluoromethyl)benzyl chloride (266 µL, 1.8 mmol), NaHCO_3_ (302 mg, 3.6 mmol) and DMF (6.0 mL). Purification: EtOAc/Hex (1:4). Yield: 207 mg, 42%. Whitish solid. mp: 160–162 °C. mp lit.^15^: 160–161 °C.

***3-(4-Fluorobenzyl)-1-(4-(trifluoromethyl)benzyl)quinazolin-2,4(1H,3H)-dione* (2).** Reagents: 3-(4-fluorobenzyl)quinazolin-2,4(*1H,3H*)-dione (1.2 mmol) obtained by method A from methyl 2-aminobenzoate and 1-fluoro-4-(isocyanatomethyl)benzene), 4-(trifluoromethyl)benzyl chloride (266 µL, 1.8 mmol), NaHCO_3_ (302 mg, 3.6 mmol) and DMF (6.0 mL). Purification: EtOAc/Hex (1:4). Yield: 102 mg, 20%. Whitish solid. mp: 142–144 °C. ^1^H NMR (300 MHz, CDCl_3_) δ 8.18 (dd, *J* = 7.9, 1.6 Hz, 1H), 7.57–7.39 (m, 5H), 7.26 (d, *J* = 8.0 Hz, 2H), 7.20–7.09 (m, 1H), 6.92 (t, *J* = 8.7 Hz, 3H), 5.34 (s, 2H), 5.21 (s, 2H). ^13^C NMR (75 MHz, CDCl_3_) δ 162.4 (d, *J_C-F_*=247 Hz), 161.5, 151.3, 139.7 (q, *J_C-F_*=2 Hz), 139.6, 135.3, 132.6 (d, *J_C-F_* = 3 Hz), 131.1 (d, *J_C-F_*= 8 Hz), 130.0 (q, *J_C-F_* = 32 Hz), 129.4, 126.7, 126.0 (q, *J_C-F_* = 4 Hz), 124.1 (q, *J_C-F_* = 272 Hz), 123.4, 115.7, 115.3 (d, *J_C-F_* = 22 Hz), 114.0, 47.0, 44.4. Anal. (C_23_H_16_F_4_N_2_O_2_) Calculated: C 64.49%, H 3.76%, N 6.54%. Found: C 64.19%, H 3.80%, N 6.49%.

***1-(4-(Trifluoromethyl)benzyl)3-(4-methoxybenzyl)quinazolin-2,4(1H,3H)-dione* (3).** Reagents: 3-(4-methoxybenzyl)quinazolin-2,4(*1H,3H*)-dione (1.2 mmol) obtained by method A from methyl 2-aminobenzoate and 1-(isocyanatomethyl)-4-methoxybenzene), 4-(trifluoromethyl)benzyl chloride (266 µL, 1.8 mmol), NaHCO_3_ (302 mg, 3.6 mmol) and DMF (6.0 mL). Purification: EtOAc/Hex (1:4). Yield: 111 mg, 21%. Whitish solid. mp: 130–132 °C. ^1^H NMR (300 MHz, DMSO-d_6_) δ 8.21–8.11 (m, 1H), 7.80–7.66 (m, 3H), 7.62–7.52 (m, 2H), 7.42–7.25 (m, 4H), 6.98–6.87 (m, 2H), 5.53 (s, 2H), 5.18 (s, 2H), 3.77 (s, 3H). ^13^C NMR (75 MHz, DMSO-d_6_) δ 161.5, 158.9, 151.3, 141.7, 139.9, 135.9, 129.8, 129.5, 128.6, 128.3 (d, *J_C-F_*=32.1 Hz), 127.6, 126.0 (q, *J* = 3.8 Hz), 124.5 (q, *J* = 272 Hz), 123.6, 122.8, 115.4 (d, *J_C-F_*=21.4 Hz), 114.1, 55.5, 46.6, 44.4. Anal. (C_24_H_19_F_3_N_2_O_3_) Calculated: C 65.45%, H 4.35%, N 6.36%. Found: C 65.26%, H 4.47%, N 5.98%.

***6-Bromo-3-(4-fluorobenzyl)-1-(4-(trifluoromethyl)benzyl)quinazolin-2,4(1H,3H)-dione* (4).** Reagents: 6-bromo-3-(4-fluorobenzyl)quinazolin-2,4(*1H,3H*)-dione (1.2 mmol) obtained by method B from 2-amino-5-bromobenzoic acid and 1-fluoro-4-(isocyanatomethyl)benzene), 4-(trifluoromethyl)benzyl chloride (191 µL, 1.5 mmol), NaHCO_3_ (302 mg, 3.6 mmol) and DMF (6.0 mL). Purification: EtOAc/Hex (1:4). Yield: 176 mg, 29%. Whitish solid. mp: 186–188 °C. ^1^H NMR (300 MHz, CDCl_3_) δ 8.29 (d, *J* = 2.4 Hz, 1H), 7.59–7.37 (m, 5H), 7.24 (d, *J* = 8.0 Hz, 2H), 6.92 (t, *J* = 8.7 Hz, 2H), 6.81 (d, *J* = 8.9 Hz, 1H), 5.31 (s, 2H), 5.19 (s, 2H). ^13^C NMR (75 MHz, CDCl_3_) δ 162.3 (d, *J_C-F_* = 247 Hz), 160.4, 150.9, 139.2 (q, *J_C-F_* = 2 Hz), 138.5, 138.1, 132.3 (d, *J_C-F_* = 2 Hz), 131.8, 131.2 (d, *J_C-F_* = 8 Hz), 130.3 (q, *J_C-F_* = 32 Hz), 126.6, 126.1 (q, *J_C-F_* = 4 Hz), 123.7 (q, *J_C-F_* = 272 Hz), 117.3, 116.4, 115.9, 115.3 (d, *J_C-F_* = 22 Hz), 47.2, 44.6. Anal. (C_23_H_15_BrF_4_N_2_O_2_) Calculated: C 54.46%, H 2.98%, N 5.52%. Found: C 54.50%, H 2.94%, N 5.51%.

***6-Bromo-1-(4-(trifluoromethyl)benzyl)-3-(4-methoxybenzyl)quinazolin-2,4(1H,3H)-dione* (5).** Reagents: 6-bromo-3-(4-methoxybenzyl)quinazolin-2,4(*1H,3H*)-dione (1.2 mmol) obtained by method B from 2-amino-5-bromobenzoic acid and 1-(isocyanatomethyl)-4-methoxybenzene), 4-(trifluoromethyl)benzyl chloride (266 µL, 1.8 mmol), NaHCO_3_ (302 mg, 3.6 mmol) and DMF (6.0 mL). Purification: EtOAc/Hex (1:4). Yield: 405 mg, 65%. Whitish solid. mp: 147–149 °C. ^1^H NMR (300 MHz, CDCl_3_) δ 8.10 (d, *J* = 8.4 Hz, 1H), 7.61 (d, *J* = 8.1 Hz, 2H), 7.50 (d, *J* = 8.7 Hz, 2H), 7.42–7.29 (m, 3H), 7.18 (d, *J* = 1.6 Hz, 1H), 6.91–6.80 (m, 2H), 5.36 (s, 2H), 5.24 (s, 2H), 3.78 (s, 3H). ^13^C NMR (75 MHz, CDCl_3_) δ 161.3, 159.6, 151.5, 140.8, 139.5 (q, *J_C-F_* = 2 Hz), 131.1, 131.1, 130.6 (q, *J_C-F_* = 32 Hz), 130.5, 129.1, 127.1, 127.1, 126.5 (q, *J_C-F_* = 4 Hz), 124.3 (q, *J_C-F_* = 272 Hz), 117.3, 115.0, 114.2, 55.6, 47.5, 45.1. Anal. (C_24_H_18_BrF_3_N_2_O_3_) Calculated: C 55.51%, H 3.49%, N 5.39%. Found: C 55.63%, H 3.55%, N 5.31%.

***7-Bromo-3-(4-fluorobenzyl)-1-(4-(trifluoromethyl)benzyl)quinazolin-2,4(1H,3H)-dione* (6).** Reagents: 7-bromo-3-(4-fluorobenzyl)quinazolin-2,4(*1H,3H*)-dione (1.2 mmol) obtained by method B from 2-amino-4-bromobenzoic acid and 1-fluoro-4-(isocyanatomethyl)benzene), 4-(trifluoromethyl)benzyl chloride (266 µL, 1.8 mmol), NaHCO_3_ (302 mg, 3.6 mmol) and DMF (6.0 mL). Purification: EtOAc/Hex (1:4). Yield: 140 mg, 22%. Whitish solid. mp: 153–155 °C. ^1^H NMR (300 MHz, CDCl_3_) δ 8.13 (d, *J* = 8.4 Hz, 1H), 7.64 (d, *J* = 8.0 Hz, 2H), 7.50–7.41 (m, 2H), 7.44–7.32 (m, 3H), 7.22 (d, *J* = 1.6 Hz, 1H), 7.02 (t, *J* = 8.7 Hz, 2H), 5.39 (s, 2H), 5.28 (s, 2H). ^13^C NMR (75 MHz, CDCl_3_) δ 162.5 (d, *J_C-F_* = 247 Hz), 160.9, 151.0, 140.5, 139.0 (q, *J_C-F_* = 2 Hz), 132.3 (d, *J_C-F_* = 2 Hz), 131.1 (d, *J_C-F_* = 8 Hz), 130.7, 130.3 (q, *J_C-F_* = 32 Hz), 130.3, 126.9, 126.7, 126.2 (q, *J_C-F_* = 4 Hz), 123.8 (q, *J_C-F_* = 272 Hz), 117.0, 115.3 (d, *J_C-F_* = 22 Hz), 114.5, 47.14, 44.59. Anal. (C_23_H_15_BrF_4_N_2_O_2_) Calculated: C 54.46%, H 2.98%, N 5.52%. Found: C 54.43%, H 3.01%, N 5.50%.

***7-Bromo-1-(4-(trifluoromethyl)benzyl)-3-(4-methoxybenzyl)quinazolin-2,4(1H,3H)-dione* (7).** Reagents: 7-bromo-3-(4-methoxybenzyl)quinazolin-2,4(*1H,3H*)-dione (1.2 mmol) obtained by method B from 2-amino-4-bromobenzoic acid and 1-(isocyanatomethyl)-4-methoxybenzene), 4-(trifluoromethyl)benzyl chloride (266 µL, 1.8 mmol), NaHCO_3_ (302 mg, 3.6 mmol) and DMF (6.0mL). Purification: EtOAc/Hex (1:4). Yield: 218 mg, 35%. Whitish solid. mp: 138–140 °C. ^1^H NMR (300 MHz, CDCl_3_) δ 8.39 (d, *J* = 2.4 Hz, 1H), 7.62 (dd, *J* = 8.2, 4.5 Hz, 3H), 7.56–7.47 (m, 2H), 7.34 (d, *J* = 8.0 Hz, 2H), 6.88 (m, 3H), 5.40 (s, 2H), 5.28 (s, 2H), 3.81 (s, 3H). ^13^C NMR (75 MHz, CDCl_3_) δ 160.4, 159.2, 151.0, 139.3 (q, *J_C-F_* = 2 Hz), 138.5, 137.9, 131.8, 130.7, 130.2 (q, *J_C-F_* = 32 Hz), 128.7, 126.6, 126.1 (q, *J_C-F_* = 4 Hz), 123.8 (q, *J_C-F_* = 272 Hz), 117.4, 116.3, 115.8, 113.8, 55.2, 47.1, 44.8. Anal. (C_24_H_18_BrF_3_N_2_O_3_) Calculated: C 55.51%, H 3.49%, N 5.39%. Found: C 55.31%, H 3.54%, N 5.30.

***6-Chloro-3-(4-fluorobenzyl)-1-(4-(trifluoromethyl)benzyl)quinazolin-2,4(1H,3H)-dione* (8).** Reagents: 6-chloro-3-(4-fluorobenzyl)quinazolin-2,4(*1H,3H*)-dione (1.2 mmol) obtained by method A from methyl 2-amino-5-chlorobenzoate and 1-fluoro-4-(isocyanatomethyl)benzene), 4-(trifluoromethyl)benzyl chloride (266 µL, 1.8 mmol), NaHCO_3_ (302 mg, 3.6 mmol) and DMF (6.0 mL). Purification: EtOAc/Hex (1:4). Yield: 55 mg, 10%. Whitish solid. mp: 160–162 °C. ^1^H NMR (300 MHz, CDCl_3_) δ 8.19 (d, *J* = 8.5 Hz, 1H), 7.62 (d, *J* = 8.1 Hz, 2H), 7.54 (dd, *J* = 8.6, 5.5 Hz, 2H), 7.34 (d, *J* = 8.0 Hz, 2H), 7.21 (dd, *J* = 8.5, 1.7 Hz, 1H), 7.05–6.95 (m, 3H), 5.37 (s, 2H), 5.26 (s, 2H). ^13^C NMR (75 MHz, CDCl_3_) δ 163.0 (d, *J_C-F_* = 247 Hz), 161.2, 151.5, 142.2, 140.9, 139.4 (q, *J_C-F_* = 2 Hz), 132.7 (d, *J_C-F_* = 3 Hz), 131.5 (d, *J_C-F_* = 8 Hz), 131.1, 130.7 (q, *J_C-F_* = 32 Hz), 127.1, 126.5 (q, *J_C-F_* = 4 Hz), 124.4, 124.1 (q, *J_C-F_* = 272 Hz), 115.7 (d, *J_C-F_* = 22 Hz), 114.5, 114.5, 47.5, 44.9. Anal. (C_23_H_15_ClF_4_N_2_O_2_) Calculated: C 59.69%, H 3.27%, N 6.05%. Found: C 59.62%, H 3.30%, N 6.01%.

***6-Chloro-1-(4-(trifluoromethyl)benzyl)-3-(4-methoxybenzyl)quinazolin-2,4(1H,3H)-dione* (9).** Reagents: 6-chloro-3-(4-methoxybenzyl)quinazolin-2,4(*1H,3H*)-dione (1.2 mmol) obtained by method A from methyl 2-amino-5-chlorobenzoate and 1-(isocyanatomethyl)-4-methoxybenzene), 4-(trifluoromethyl)benzyl chloride (266 µL, 1.8 mmol), NaHCO_3_ (302 mg, 3.6 mmol) and DMF (6.0 mL). Purification: EtOAc/Hex (1:4). Yield: 387 mg, 71%. Whitish solid. mp: 145–147 °C. ^1^H NMR (300 MHz, CDCl_3_) δ 8.21 (d, *J* = 8.4 Hz, 1H), 7.64 (d, *J* = 8.1 Hz, 2H), 7.58–7.47 (m, 2H), 7.37 (d, *J* = 8.0 Hz, 2H), 7.22 (dd, *J* = 8.5, 1.7 Hz, 1H), 7.02 (d, *J* = 1.7 Hz, 1H), 6.91–6.83 (m, 2H), 5.39 (s, 2H), 5.27 (s, 2H), 3.81 (s, 3H). ^13^C NMR (75 MHz, CDCl_3_) δ 160.8, 159.2, 151.2, 141.6, 140.5, 139.1 (q, *J_C-F_* = 2 Hz), 130.7, 130.2 (q, *J_C-F_* = 32 Hz), 128.8, 126.7, 126.1 (q, *J_C-F_* = 4 Hz), 124.0 (q, *J_C-F_* = 272 Hz), 123.9, 114.3, 114.0, 113.8, 55.2, 47.1, 44.7. Anal. (C_24_H_18_ClF_3_N_2_O_3_) Calculated: C 60.70%, H 3.82%, N 5.90%. Found: C 60.72%, H 3.80%, N 5.86%.

***3-(4-Fluorobenzyl)-1-(4-(trifluoromethyl)benzyl)-6,7-dimethoxyquinazolin-2,4(1H,3H)-dione* (10).** Reagents: 3-(4-fluorobenzyl)-6,7-dimethoxyquinazolin-2,4(*1H,3H*)-dione (1.2 mmol) obtained by method A from methyl 2-amino-4,5-dimethoxibenzoate and 1-fluoro-4-(isocyanatomethyl)benzene), 4-(trifluoromethyl)benzyl chloride (266 µL, 1.8 mmol), NaHCO_3_ (302 mg, 3.6 mmol) and DMF (6.0 mL). Purification: EtOAc/Hex (1:2). Yield: 73 mg, 15%. Whitish solid. mp: 180–182 °C. ^1^H NMR (300 MHz, CDCl_3_) δ 7.62 (m, 3H), 7.57 (dd, *J* = 8.6, 5.5 Hz, 2H), 7.38 (d, *J* = 8.0 Hz, 2H), 7.02 (t, *J* = 8.7 Hz, 2H), 6.43 (s, 1H), 5.42 (s, 2H), 5.30 (s, 2H), 3.93 (s, 3H), 3.77 (s, 3H). ^13^C NMR (75 MHz, CDCl_3_) δ 162.7 (d, *J_C-F_* = 246 Hz), 161.5, 155.6, 151.9, 146.2, 140.3 (q, *J_C-F_* = 2 Hz), 135.6, 133.2 (d, *J_C-F_* = 3 Hz), 131.4 (d, *J_C-F_* = 8 Hz), 130.6 (q, *J_C-F_* = 32 Hz), 127.1, 126.4 (q, *J_C-F_* = 4 Hz), 124.0 (q, *J_C-F_* = 272 Hz), 115.6 (d, *J_C-F_* = 22 Hz), 109.6, 108.5, 97.4, 56.7, 56.5, 47.7, 44.8. Anal. (C_25_H_20_F_4_N_2_O_4_) Calculated: C 61.48%, H 4.13%, N 5.74%. Found: C 60.67%, H 4.11%, N 5.68%.

***1-(4-(Trifluoromethyl)benzyl)-6,7-dimethoxy-3-(4-methoxybenzyl)quinazolin-2,4(1H,3H)-dione* (11).** Reagents: 6-7-dimethoxy-3-(4-methoxybenzyl)quinazolin-2,4(*1H,3H*)-dione (1.2 mmol) obtained by method A from methyl 2-amino-4,5-dimethoxibenzoate and 1-(isocyanatomethyl)-4-methoxybenzene), 4-(trifluoromethyl)benzyl chloride (266 µL, 1.8 mmol), NaHCO_3_ (302 mg, 3.6 mmol) and DMF (6.0 mL). Purification: EtOAc/Hex (1:2). Yield: 72 mg, 12%. Whitish solid. mp: 171–173 °C. ^1^H NMR (300 MHz, CDCl_3_) δ 7.55 (s, 1H), 7.52 (d, *J* = 8.0 Hz, 2H), 7.44 (d, *J* = 8.6 Hz, 2H), 7.28 (d, *J* = 8.0 Hz, 2H), 6.78 (d, *J* = 8.7 Hz, 2H), 6.32 (s, 1H), 5.32 (s, 2H), 5.19 (s, 2H), 3.83 (s, 3H), 3.70 (s, 3H), 3.67 (s, 3H). ^13^C NMR (75 MHz, CDCl_3_) δ 161.6, 159.5, 155.5, 151.9, 146.2, 140.5 (q, *J_C-F_* = 2 Hz), 135.6, 131.0, 130.5 (q, *J_C-F_* = 32 Hz), 129.7, 127.1, 126.4 (q, *J_C-F_* = 4 Hz), 124.3 (q, *J_C-F_* = 272 Hz), 114.1, 109.6, 108.6, 97.4, 56.6, 56.5, 55.6, 47.6, 45.0. Anal. (C_26_H_23_F_3_N_2_O_5_) Calculated: C 62.40%, H 4.63%, N: 5.60%. Found: C 62.09%, H 4.56%, N: 5.52%.

***8-Bromo-3-(4-fluorobenzyl)-1-(4-(trifluoromethyl)benzyl)-6-methylquinazolin-2,4(1H,3H)-dione* (12).** Reagents: 8-bromo-3-(4-fluorobenzyl)-6-methylquinazolin-2,4(*1H,3H*)-dione (1.2 mmol) obtained by method B from 2-amino-3-bromo-6-methylbenzoic acid and 1-fluoro-4-(isocyanatomethyl)benzene), 4-(trifluoromethyl)benzyl chloride (266 µL, 1.8 mmol), NaHCO_3_ (302 mg, 3.6 mmol) and DMF (6.0 mL). Purification: EtOAc/Hex (1:4). Yield: 106 mg, 17%. Whitish solid. mp: 183–185 °C. ^1^H NMR (300 MHz, CDCl_3_) δ 8.00 (d, *J* = 2.2 Hz, 1H), 7.62 (d, *J* = 2.2 Hz, 1H), 7.47 (d, *J* = 8.1 Hz, 2H), 7.38 (dd, *J* = 8.6, 5.5 Hz, 2H), 7.17 (d, *J* = 8.0 Hz, 2H), 6.88 (t, *J* = 8.7 Hz, 2H), 5.66 (s, 2H), 5.11 (s, 2H), 2.29 (s, 3H). ^13^C NMR (75 MHz, CDCl_3_) δ 162.3 (d, *J_C-F_* = 247 Hz), 160.7, 152.2, 142.9, 141.7 (q, *J_C-F_* = 2 Hz), 137.2, 135.2, 132.2 (d, *J_C-F_* = 2 Hz), 131.1 (d, *J_C-F_* = 8 Hz), 129.5 (q, *J_C-F_* = 32 Hz), 129.2, 126.5, 125.4 (q, *J_C-F_* = 4 Hz), 124.0 (q, *J_C-F_* = 272 Hz), 119.5, 115.2 (d, *J_C-F_* = 22 Hz), 107.5, 51.3, 44.6, 20.0. Anal. (C_24_H_17_BrF_4_N_2_O_2_) Calculated: C 55.35%, H 3.29%, N 5.37%. Found: C 55.35%, H 3.35%, N 5.35%.

***8-Bromo-1-(4-(trifluoromethyl)benzyl)-3-(4-methoxybenzyl)-6-methylquinazolin-2,4(1H,3H)-dione* (13).** Reagents: 8-bromo-3-(4-methoxybenzyl)-6-methylquinazolin-2,4(*1H,3H*)-dione (1.2 mmol) obtained by this method: In round‐bottomed flask with a magnetic stir bar, 2-amino-3-bromo-5-methylbenzoic acid (1.0 equiv.) is dissolved in THF (5 mL/mmol) and 1.0 equiv. of 1-(isocyanatomethyl)-4-methoxybenzene is added to the mixture, which is stirred in an oil bath at 80 °C overnight. The solvent is rotary evaporated and replaced with EtOH (20 mL/mmol). Then, 2 mL/g of concentrated HCl (12.1 M) is added and the mixture is reheated to 70 °C until complete conversion to the 3‐substituted quinazolinedione (3 h). The reaction mixture is cooled in an ice bath and water (20 mL/g) is added, which causes a precipitate. This slurry is stirred for 30 min, filtered, washed with water (10 mL/mmol), and then heptanes (3 × 10 mL/mmol) before drying the solids under a stream of nitrogen); 4-(trifluoromethyl)benzyl chloride (266 µL, 1.8 mmol), NaHCO_3_ (302 mg, 3.6 mmol) and DMF (6.0 mL). Purification: EtOAc/Hex (1:4). Yield: 204 mg, 32%. Whitish solid. mp: 158–160 °C. ^1^H NMR (500 MHz, CDCl_3_) δ 8.10 (dd, *J* = 2.3, 0.9 Hz, 1H), 7.71 (dd, *J* = 2.3, 0.8 Hz, 1H), 7.57 (d, *J* = 8.1 Hz, 2H), 7.50–7.40 (m, 2H), 7.34–7.23 (m, 2H), 6.84 (d, *J* = 8.7 Hz, 2H), 5.76 (s, 2H), 5.19 (s, 2H), 3.80 (s, 3H), 2.39 (s, 3H). ^13^C NMR (125 MHz, CDCl_3_) δ 161.1, 159.6, 152.6, 143.2, 142.2, 137.6, 135.5, 131.0, 129.6 (q, *J_C-F_* = 32 Hz), 129.6, 129.1, 126.9, 125.8 (q, *J_C-F_* = 4 Hz), 124.4 (q, *J_C-F_* = 272 Hz), 120.0, 114.1, 107.8, 55.6, 51.6, 45.2, 20.4. Anal. (C_25_H_20_BrF_3_N_2_O_3_) Calculated: C 56.30%, H 3.78%, N 5.25%. Found: C 56.20%, H 3.76%, N 5.18%.

### Microsomal stability assays

Mouse or human liver microsomes (S9), reduced nicotinamide adenine dinucleotide phosphate (NADPH) generating system solutions and uridine glucuronosyltransferase (UGT) reaction mix (BD Biosciences) were kept at −80 °C. The test compounds and reference compound diclofenac were formulated in DMSO at 10 µM. The assay was carried out based on the BD Biosciences Guidelines for Use (TF000017 Rev1.0) with minor adaptations. The metabolic stability of the compounds was studied through the CYP_450_ superfamily (Phase-I metabolism) by fortification with NADPH and through UGT enzymes (Phase-II metabolism) by fortification with uridine diphosphate glucuronic acid (UDPGA). For CYP_450_ and other NADPH dependent enzymes, the compounds were incubated at 5 µM together with 0.5 mg/mL S9 in potassium phosphate buffer in a reaction started by the addition of 1 mM NADP. At defined time points, 20 µL was withdrawn from the reaction mixture and 80 µL cold acetonitrile (ACN) was added to inactivate the enzymes and precipitate the protein. The mixture was vortexed for 30 s and centrifuged at 4 °C for 5 min to collect the supernatant. For UGT enzymes, the compounds were incubated at 5 µM together with 0.5 mg/mL S9 in a reaction started by the addition of 2 mM UDPGA cofactor. The loss of parent compound was determined using liquid chromatography (UPLC) (Waters Aquity^TM^) coupled with tandem quadrupole mass spectrometry (MS^2^) (Waters Xevo^TM^), equipped with an electrospray ionisation (ESI) interface and operated in multiple reaction monitoring (MRM) mode.

### *In vivo* antischistosomal efficacy

PZQ was obtained from Egyptian International Pharmaceutical Industries Company (EIPICO) and aminobenzotriazole (ABT) from Fluorochem Ltd.

#### Experimental animals

Male Swiss albino mice (CD-1) obtained from SBSC and weighing 18–20 g were housed under environmentally controlled room temperature of 20–22 °C, 12 h light/dark cycle and 50–60% humidity with food and water *ad libitum* throughout the acclimatisation and experimental periods. All the animal experiments were conducted in accordance with the Guide for Care and Use of Laboratory Animals and were approved by the Institutional Review Board of Theodor Bilharz Research Institute (TBRI).

#### Infection and experimental design

Mice were infected with *S. mansoni* cercariae [provided by Schistosome Biology Supply Center (SBSC)] using body immersion^16^ by exposure to 80 ± 10 cercariae/mouse. Infected mice were divided into six groups: groups 1 and 2 were treated the vehicle and the CYP_450_ inhibitor ABT respectively; groups 3 and 4 were dosed for 5 days with NPD-1246 at 20 and 10 mg/kg, respectively, while group 5 was treated with PZQ at 10 mg/kg/day for 5 days. Group 6 was treated with NPD-1246 combined with PZQ, each at 10 mg/kg/day for 5 days starting from week 7 post-infection. To minimise first-pass elimination, ABT was administered at 100 mg/kg/day for 5 days 2 h prior to each compound administration. NPD-1246 and PZQ were freshly suspended in 2% Cremophore-EL (Sigma-Aldrich). All drug administrations were performed orally.

#### Parasitological criteria for cure

Ten days post-treatment, all mice were sacrificed and perfused, and the number of worms recovered (worm burden) was quantified and sexed^17^. The number of eggs per gram of liver or intestinal tissue was counted^18^. The percentage of egg developmental stages (oogram pattern) was studied^19^ in which eggs at different stages of maturity (from I to IV) were identified and counted. Mature eggs and dead eggs (granular, dark, and semi-transparent) were also counted in three fragments of intestine and the mean number of each stage was calculated.

#### Statistical analysis

The percentage reduction of worm or egg burden in each treated group was calculated. The 50% effective concentration (EC_50_) was calculated using Prism (GraphPad; Version 5.0) software using a variable slope for the sigmoidal curve with an upper limit of 100%. Results are expressed as mean ± SEM. A two-tailed, unpaired Student’s *t*-test was used to detect the significance of difference between the means of different groups. Results are considered significant if *p* value is <0.05.

#### *In vitro S. mansoni* worm killing

Stock solutions of 5 mM PZQ and the quinazoline derivatives **2**–**13** were prepared in DMSO. Concentrations of 100 µM, 50 µM, 25 µM, 10 µM and 5 µM were freshly prepared on the day of experiment in RPMI-1640 medium. All compounds were initially tested at 100 and 50 µM; those showing worm killing were further tested at 25, 10 and 5 µM.

Worms were obtained from SBSC of TBRI. Six to eight worms were placed in 12-well tissue culture plates and fresh RPMI-1640 medium (supplemented with glutamine, 20% newborn calf serum and streptomycin, penicillin, and gentamicin), containing the indicated concentration of the test compound was added^20^^,^^21^. Worms were incubated overnight in a CO_2_ incubator at 37 °C. On the 2nd day, worms were examined by microscopy, washed three times with normal saline, fresh medium was added and the incubation was continued. On the 3rd day, worm motility was observed and on the 4th day, medium was changed again. On day 5 (end of the observation period), worms were microscopically examined for their motility and appearance. Each concentration was tested in duplicate, and the final recording of percent worm mortality was determined as the number of dead worms [contracted and opaque] divided by the total number of worms × 100. Negative controls used medium without additions or medium with 2% DMSO; positive control media containing identical concentrations of PZQ were tested in parallel.

#### *S. mansoni* ovipositing capacity

12-well tissue culture plates were used, each well containing 6–8 worms with at least one worm couple. Worms were incubated overnight in a CO_2_ incubator at 37 °C. Each concentration was tested in duplicate wells. On day 4, eggs were counted and discarded and the medium was changed. On day 5 (end of observation period) newly deposited eggs were counted. The final egg number is the total count of days 4 and 5 for each concentration tested and the final recording of the percentage of egg reduction was determined as [the total number of eggs on days 4 and 5 of control – the total number of eggs on days 4 and 5 of treated]/the total number of eggs on days 4 and 5 of control × 100.

### Metabolic stability prediction

To determine the most likely sites of CYP_450_ mediated metabolism of NPD-1246, SMARTCyp^22^ was applied using the default settings. This tool determines the sites in a molecule that are susceptible to be metabolised using the 2D structure of the compound. It is based on a model that predicts the reactivity at C, S, N and P positions in a given ligand based on a series of over 40 rules derived from quantum chemical and calculations of energies required for oxidation using density functional theory (DFT). Finally, the atoms in the molecule are ranked according to these results.

### Target prediction

With the objective of searching a potential MOA for this family of compounds, a target prediction study was performed. A consensus methodology was applied considering several approaches that involve ligand-based target prediction. Three different strategies were selected as representatives of the different tools available. Among them, polypharmacology browser (PPB)^23^ was used using all the fingerprints combinations available in the tool and default parameters. This technique was selected because it applies an important number of fingerprints alone and in combination. Also, similarity ensemble approach (SEA) was selected^24^^,^^25^ since both tools search in databases such as ChEMBL with an impressive amount of biological data to retrieve the most accurate results. These methods were used using default settings. From the list of potential targets, the first 35 were further considered. Finally, a search on the PDB using the main scaffold of the compounds was carried out to look for already crystallised structures.

### Homology modelling

In order to produce the most accurate model for *S. mansoni* aldose reductase (G4LXS0), a template search was performed using the Swiss-Model server^26^. In the next step, the sequential alignment of the target protein was carried out with the different templates selected, the human aldose reductase (P15121) and *S. japonicum* Q5DD64 sequences. The templates and target sequences were retrieved from Uniprot database^27^.

Due to the high identity with the *S. japonicum* protein, this template was selected to further build the target model using the Swiss-Model server. Once the model was built, the estimation of the protein model accuracy is required. For that purpose, model quality assessment was performed using different metrics. The local composite scoring function QMEAN (Qualitative Model Energy Analysis) allows discriminating good from bad models assessing geometrical aspects of the protein structure using several statistical descriptors. Energetic and geometric quality assessment was also performed using Ramachandran plot^28^, ProSA^29^, ERRAT^30^ or VERIFY3D^31^ tools.

### Docking studies

#### Ligand preparation

The preparation of the test compounds and the 2D-to-3D conversion was carried out using the LigPrep tool^32^, a module of the Schrödinger software package. This tool allows the preparation of molecules including different steps such as the calculation of the ionisation state of the molecules at a pH range, the addition of hydrogen atoms, and a final energy minimisation using the OPLS-2005 force field^33^^,^^34^. To perform the studies, physiological pH conditions were used to prepare the molecules, all of them were desalted and in the last step, the compounds were minimised as default.

#### Protein preparation

The structures of the proteins used in this study were preprocessed and refined using the Protein Preparation Wizard tool^35^^,^^36^ included on Maestro^37^. H-bond assignment and calculation of the protonation state of the residues at physiological pH with a final restraint minimisation were carried out.

#### Docking studies

Automated docking protocol was used to assess the appropriate binding mode and suitable poses of the reference compound and the ligand. A Lamarckian genetic algorithm^38^ method implemented in the programme AutoDock 4.2^39^ was applied. For docking calculations, Gasteiger charges were added, rotatable bonds were set by AutoDock tools (ADT) and all torsions were allowed to rotate for the ligand. In all the cases, we used grid maps with a grid box size of 60 × 60 × 60 Å^3^ points and a grid-point spacing of 0.375 Å, using as centroid of the grid the key Trp111 presents in the catalytic site. The docking protocol consisted of 200 independent genetic algorithm runs, population size of 150 and maximum number of evaluation 250,000, while the remaining parameters were conserved as default. Final best-docked poses were grouped into clusters, within the default 2.0 Å RMSD. Best energetic and most representative docking clusters were analysed by visual inspection according to the binding energies and relative population provided by the software. Best poses in these clusters were considered as most reliable representatives of the ligand-binding mode and were further studied.

In the case of *S. mansoni* aldose reductase an additional strategy was carried out to optimise the structure of the model built. This strategy is called induced fit docking (IFD)^40^^,^^41^ and is based on fitting the ligand to the protein binding sites allowing changes in the residues geometry, mainly in their side chain orientations. First, Prime^42^ predicts the active site structure using the pose of compound NPD-1246 to rearrange nearby side chains of the protein and minimising the overall energy of the protein. Finally, each ligand is re-docked into its corresponding low energy protein structures and the resulting complexes are ranked according to docking score. Extra precision (XP) mode was used in a standard protocol and no constraints were set, residues were optimised to 5.0 Å of the ligand poses and the rest of the parameters were set as default.

## Results

### *In vitro* metabolic stability

NPD-1246 was exposed to mouse S9 microsomal fractions to investigate the *in vitro* metabolic stability through phase-I and phase-II metabolism (Table 1). The results indicate that the compound becomes extensively metabolised through phase-I but not through phase-II metabolism. After 30 min, only 27% of parent compound is left indicating poor metabolic stability. The reference drug diclofenac showed extensive phase-I and phase-II metabolism, validating the performance of the assay.

**Table 1.** *In* *vitro* metabolic stability of NPD-1246: percentage of parent compound remaining over time in the presence of mouse liver microsomes.

% Parent compound remaining upon incubation					
Phase I/II	Time (min)	NPD-1246		Diclofenac	
		Average	SD	Average	SD
CYP_450_-NADPH	0	100	−	100	−
	15	66	5	87	2
	30	27	9	70	6
	60	8	2	48	1
		(*n* = 2)		(*n* = 2)	
UGT enzymes	0	100	−	100	−
	15	111	4	41	1
	30	111	2	44	8
	60	106	5	34	2
		(*n* = 2)		(*n* = 2)	

### Activity in the* S. mansoni*-infected mouse model

NPD-1246 was previously shown to have relevant schistosomicidal activity potential *in vitro*^14^. Since NPD-1246 proved to be metabolically unstable (Table 1), the compound was administered orally concomitantly with the CYP_450_ inhibitor ABT to counter metabolic degradation^43^. Infected mice were treated either alone with NPD-1246 or in combination with PZQ. Control groups receiving ABT or ABT + PZQ were included as well.

**Figure 2.**
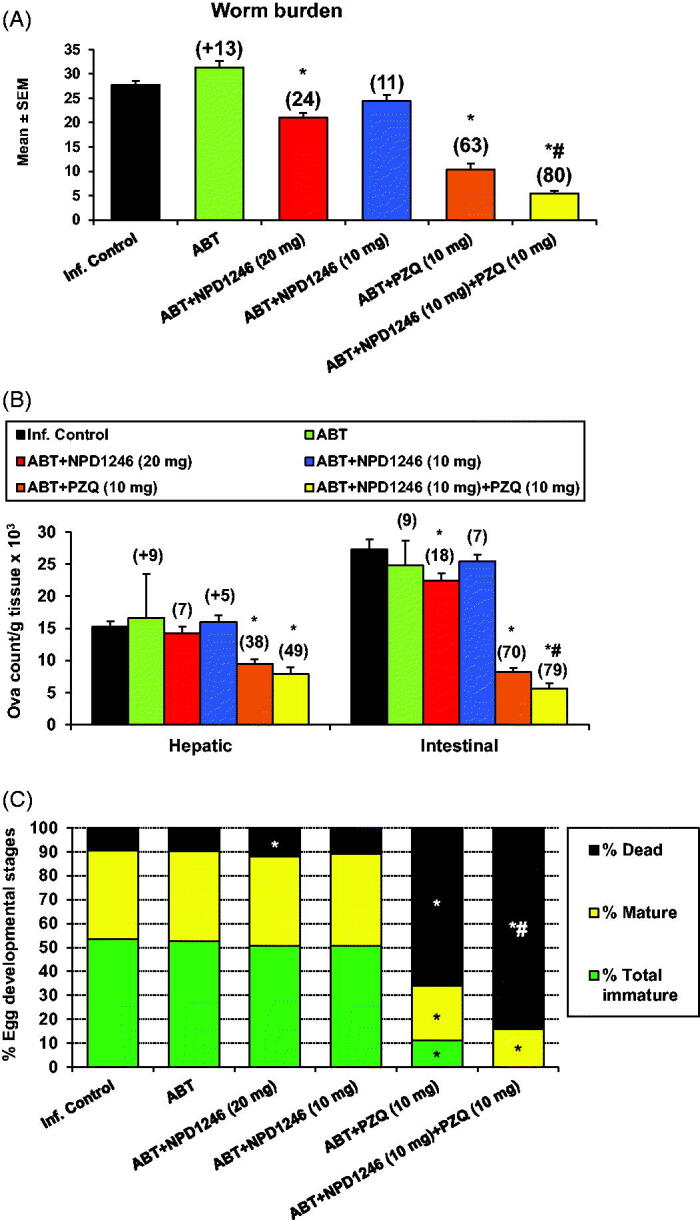
Effect of NPD-1246 alone (in a dose of 20 or 10 mg/kg/day) or in combination with PZQ (10 mg/kg/day each) for 5 days treatment on (A) worm burden, (B) tissue egg load and (C) oogram pattern in *S. mansoni*-infected mice sacrificed 10 days post end of treatment. *Significantly different from infected control at *p* < 0.05. ^#^Significantly different from PZQ group at *p* < 0.05. Numbers above columns and between parentheses represent percentage change from infected control group.

The pooled data of two experiments (number of mice ranged from 6 to 10/group in each experiment) upon a 5-day treatment with NPD-1246 at 20 mg/kg revealed a reduction in total worm burdens and intestinal tissue egg load by 24% and 18%, respectively (Figure 2(A,B)), accompanied with significant increase in the percentage of dead eggs when compared to the infected untreated group (Figure 2(C)). Administration of NPD-1246 at 10 mg/kg did not produce any significant change in these parameters with respect to the group dosed at 20 mg/kg (Figure 2). Treatment with PZQ at 10 mg/kg significantly reduced total worms by 63% and hepatic and intestinal tissue egg loads by 38% and 70%. Total immature and mature eggs were also reduced with a significant increase in dead eggs. Finally, 5-day co-treatment of NPD-1246 and PZQ at 10 mg/kg/day revealed an enhanced reduction in total worms (80% vs. 63% for PZQ alone), intestinal tissue egg load (79% vs. 70% for PZQ alone) with complete disappearance of immature eggs and increase of dead eggs (84% vs. 66% for PZQ alone).

### Metabolic stability prediction

**Figure 3.**
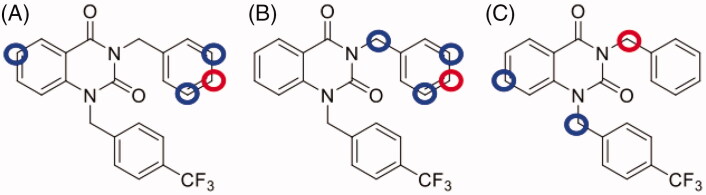
Metabolic site prediction using SMARTCyp web server for NPD-1246. Top-ranked sites (red circles) and minor sites (blue circles) predicted to be metabolised by (A) CYP_2C9_, (B) CYP_2D6_ and (C) CYP_3A4_.

NPD-1246 shows promising antischistosomal activity potential *in vivo*, but was found metabolically unstable. A medicinal chemistry programme was therefore designed with the aim of improving its drug-like properties that would enable further development. Computational studies using SmartCyp^22^ were performed to identify the positions potentially susceptible for metabolic degradation. This software tool predicts CYP_3A4_, CYP_2D6_ and CYP_2C9_ effect on the target molecule and ranks atoms according to their probability to be modified by metabolism (Supplementary Figures S1–S3). Several potential sites were identified (Figure 3). The most important of which are C6 in the quinazoline scaffold and the *meta*- and *para*-positions of the benzyl substituent in the N1.

### Design and synthesis of NPD-1246 derivatives

**Scheme 1.**
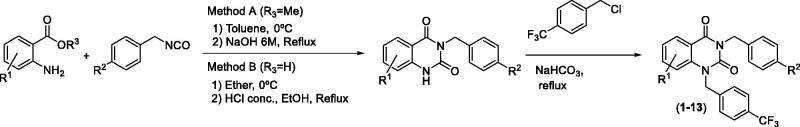
Synthesis of 3-benzyl-1-(4-(trifluoromethyl)benzyl)quinazolin-2,4(*1H*,*3H*)-diones **1**–**13**.

With the aim of increasing the metabolic stability of 3-benzyl-1-((4-trifluoromethyl)benzyl)quinazolin-2,4(*1H*,*3H*)-dione (NPD-1246, **1**), different chemical modifications were prioritised to block the predicted positions. Methoxy and fluor were chosen as *p*-substituents for the benzyl tail attached to N3, while the benzene fused ring from the quinazoline remained without substituents or with halogen or alkyl groups attached to different positions. The synthesis was accomplished in two stages following previously described procedures^15^. In the first step, the reaction between the corresponding benzyl isocyanate and the anthranilic acid derivative yielded the substituted 3-benzylquinazolin-2,4(*1H*,*3H*)-dione used in the next step without further purification. In a second step, the mono-substituted quinazoline reacts with 4-(trifluoromethyl)benzyl chloride in basic media to obtain the desired di-substituted quinazolines **1**–**13** with moderate yields (Scheme 1). The compounds were characterised based on the analytical data detailed in the chemical procedures on the experimental part.

### *In vitro* activity against S. mansoni

The new quinazolines **2**–**13** were initially tested at 100 and 50 µM against male and female worms and compared with the previously reported data for parent compound NPD-1246^14^. The parameters for antischistosomal activity were worm mortality, motor activity alterations (sluggish worm movement or spastic contractions), unpairing and absence or reduction in egg numbers (Table 2). While almost all the new compounds are able to reduce the egg numbers and to separate or insult the coupling at both concentrations, **9** and **10** revealed 100% worm killing at 100 µM and 29% and 93% at 50 µM, respectively. Based on these data, both compounds were selected for further dose-titration. The EC_50_ for **9** was comparable to NPD-1246 (**1**) (47 µM vs. 50 µM) with **10** being slightly more active (EC_50_ 25 µM) (Table 3). These values were obtained taking both males and females into account. When analysing each sex separately, the results remain similar pointing out that there are no obvious sex differences (Table 4). As for coupling and ova production, both compounds present a similar behaviour to NPD-1246 showing a significant reduction in egg numbers at 5 µM (i.e. the lowest concentration tested).

**Table 2.** Mature worm killing and ovipositing at 100 µM and 50 µM of new quinazolines (**2**–**13**) in comparison with previously reported data for NPD-1246 (**1**)^14^.


			Worm killing^a^ (% of total)		Uncoupling		Reduction in number of eggs (%)	
R^1^	R^2^		100 µM	50 µM	100 µM	50 µM	100 µM	50 µM
H	H	NPD-1246 **(1)**	100	53	Yes	Yes	100	100
H	F	**2**	100	0	Yes	No	100	100
H	OMe	**3**	75	0	Yes	Yes	100	100
6-Br	F	**4**	100	13	Yes	Yes	100	100
6-Br	OMe	**5**	100	0	Yes	No	100	31
7-Br	F	**6**	88	0	Yes	Yes	100	100
7-Br	OMe	**7**	0	0	No	No	50	35
6-Cl	F	**8**	100	0	Yes	Yes	100	100
6-Cl	OMe	**9**	100	29	Yes	Yes	100	100
6,7-diOMe	F	**10**	100	93	Yes	Yes	100	100
6,7-diOMe	OMe	**11**	86	0	Yes	Yes	100	100
6-Me,8-Br	F	**12**	0	0	Yes	No	100	50
6-Me,8-Br	OMe	**13**	33	0	Yes	Yes	100	100

^a^The final recording of worm killing was determined on day 5 (end of the observation period).

**Table 3.** Mature (6 weeks old) worm killing and ovipositing under different concentrations of selected compounds in comparison with NPD-1246 (**1**) data^14^ previously reported.

	Worm killing^a^ (% of total)					EC_50_	Uncoupling					Reduction in number of eggs (%)				
	100 µM	50 µM	25 µM	10 µM	5 µM	(µM)	100 µM	50 µM	25 µM	10 µM	5 µM	100 µM	50 µM	25 µM	10 µM	5 µM
NPD-1246 **(1)**	100	53	0	0	0	50	Yes	Yes	Yes	No	No	100	100	100	20	10
**9**	100	88	0	0	0	47	Yes	Yes	Yes	No	No	100	100	100	73	67
**10**	100	100	50	8	0	25	Yes	Yes	Yes	No	No	100	100	100	80	77

^a^The final recording of worm killing was determined on day 5 (end of the observation period).

**Table 4.** Mature (male & female) worm killing under different concentrations of selected compounds in comparison with NPD-1246 (**1**) data^14^ previously reported.

	Worm killing^a^ (male worms)					EC_50_	Worm killing^a^ (female worms)					EC_50_
	100 µM	50 µM	25 µM	10 µM	5 µM	(µM)	100 µM	50 µM	25 µM	10 µM	5 µM	(µM)
NPD-1246 **(1)**	100	50	0	0	0	50	100	57	0	0	0	50
**9**	100	100	0	0	0	41	100	75	0	0	0	49
**10**	100	100	63	14	0	20	100	100	33	0	0	26

^a^The final recording of worm killing was determined on day 5 (end of the observation period).

### *In vitro* metabolic stability

The stability of **9** and **10** through phase-I and phase-II metabolism by mouse microsomes was investigated in a similar way than for NPD-1246 (Table 5). Both compounds presented a better profile than NPD-1246 with 51% and 39% of **9** and **10** remaining after 30 min, hence indicating acceptable stability. With human microsomes, the stability was even better with 61% and 83% of **9** and **10**, respectively remaining after 30 min. None of the compounds was affected by phase-II metabolism.

**Table 5.** *In vitro* metabolic stability of **9** and **10**: percentage of parent compound remaining over time in the presence of mouse and human liver microsomes.

			% Parent compound remaining upon incubation					
Microsomes	Phase I/II	Time (min)	**9**		**10**		Diclofenac	
			Average	SD	Average	SD	Average	SD
Mouse	CYP_450_-NADPH	0	100	–	100	–	100	–
		15	91	3	85	5.3	61	
		30	51	3.3	39	0.55	34	
		60	44	0.45	32	0.43	27	
			(*n* = 2)				(*n* = 1)	
	UGT enzymes	0	100	–	100	–	100	
		15	101	2.6	89	8.3	34	
		30	103	3.6	83	8.7	32	
		60	105	0.8	91	18.3	30	
			(*n* = 2)		(*n* = 2)		(*n* = 1)	
Human	CYP_450_-NADPH	0	100	–	100	–	100	–
		15	104	4.9	108	3.7	27	
		30	61	2.4	83	18.8	5	
		60	67	3.2	65	1.3	1	
			(*n* = 2)		(*n* = 2)		(*n* = 1)	
	UGT enzymes	0	100	–	100		100	
		15	94	27.0	102	2.4	11	
		30	95	12.9	106	4.5	9	
		60	97	14.1	98	1.8	9	
			(*n* = 2)		(*n* = 2)		(*n* = 1)	

### Computational target deconvolution

Based on the confirmed *in vitro* and *in vivo* activity potential of the quinazoline family against *S. mansoni*, *in silico* studies were conducted to obtain additional information about their putative MOA. A consensus *in silico* methodology using tools based on ligand similarity was used, relying on the general assumption that related molecules will have similar activity and interaction patterns. Firstly, the PPB^23^ was used as an exhaustive method in terms of fingerprint similarity. Secondly, the SEA^24^^,^^25^ was selected to identify targets based on a group of known compounds rather than a single compound. As a third strategy, a structural comparison searching for similar structures in terms of chemical scaffold that had already been crystallised as protein inhibitors was performed using the protein data bank (PDB)^44^. The three methodologies were sequentially applied to the reference compound NPD-1246.

**Figure 4.**
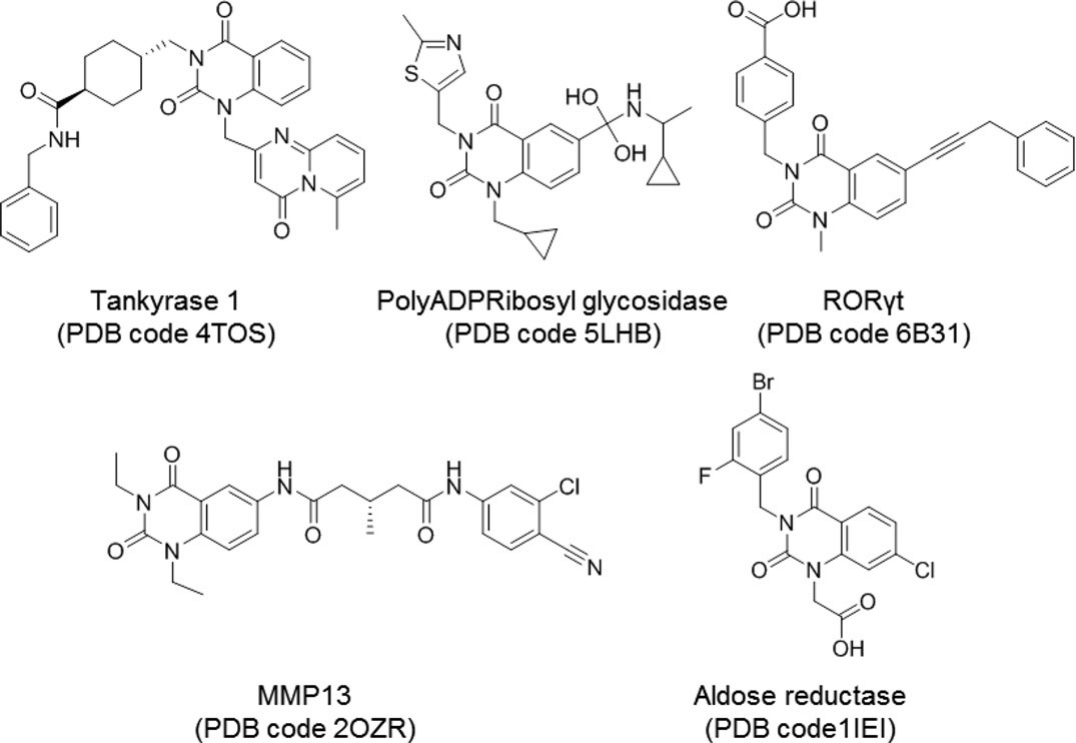
Quinazoline-related structures crystallised with different target proteins according to the scaffold search in the PDB (access codes included).

The PPB search provides a list of potential targets, organisms and cell lines where a compound may have biological activity. In this study, the cell lines and organisms were discarded and only targets involving proteins and/or enzymes were conserved. The 35 top-ranked results are collected in Supplementary Table S1. The second approach using the SEA tool produced a ranking of targets, from which the top 35 were taken into account (Supplementary Table S2). Finally, the chemical scaffold search in the PDB retrieved five different results of crystallised quinazoline derivatives with different enzymes (Figure 4).

With the three methodologies combined one common target was revealed, namely the human aldose reductase. Moreover, one compound crystallised with this enzyme, zenarestat (PDB code 1IEI)^45^ (Figure 4) retrieved in the search is a di-substituted quinazoline similar to NPD-1246.

### Homology modelling

**Figure 5.**
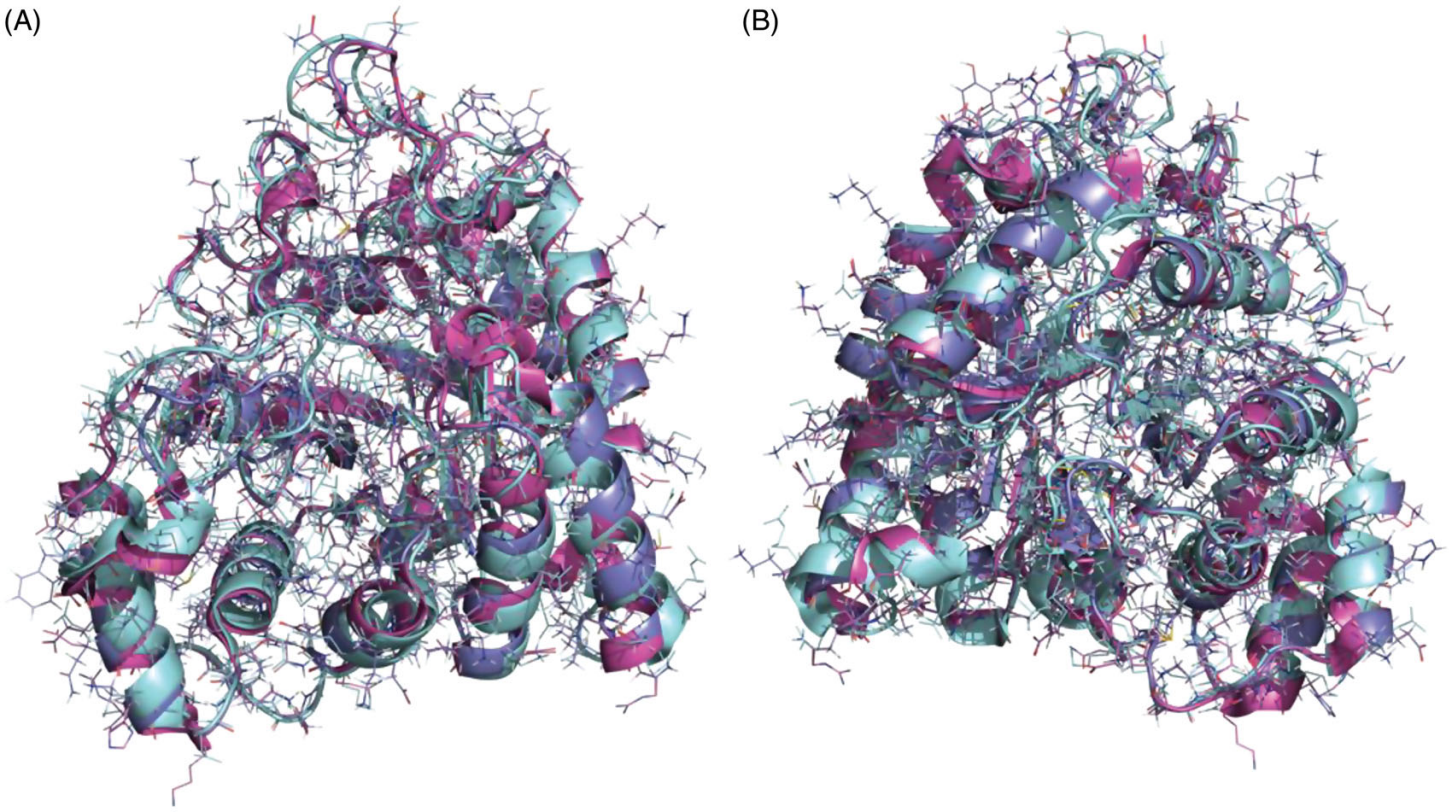
Superposition of the crystal structures of human aldose reductase 1IEI, depicted in cyan, *S. japonicum* aldose reductase 4HBK in magenta and the homology model of *S. mansoni* aldose reductase in purple, (A) front view and (B) back view.

As the crystal structure of aldose reductase in *S. mansoni* was not available at the time of the study, a search for homologous structures was done to build an accurate model that would guide us in the computational studies on the potential binding mode of our compounds in the enzyme. The closest homologue with a crystal structure available is the aldose reductase of *S. japonicum* (PDB code 4HBK)^46^ and a sequence alignment was carried out (Supplementary Figure S4). The sequences of the *Schistosoma spp* are almost identical showing an identity of 83.23% but the identity between the human and the *S. mansoni* proteins is 50.32%. The similarity between the *Schistosoma* enzymes is even higher considering that most of the mutations are related amino acids with similar properties. For this reason, the structure of aldose reductase from *S. japonicum* was chosen as the most accurate template to obtain a reliable model of our target protein using the Swiss-Model server^26^. The final structure was validated from a geometric and energetic point of view using several widely used metrics (Supplementary Table S3). More than 98% of the torsion angles are in allowed regions of the Ramachandran plot^28^. It also presents an overall quality factor over 93.3% for ERRAT^30^ parameter and 92.9% according to the verify analysis^47^. Superposition of the crystal structures of human aldose reductase (PDB code 1IEI), *S. japonicum* aldose reductase (PDB code 4HBK) and the homology model of *S. mansoni* aldose reductase is depicted in Figure 5.

### Docking studies

**Figure 6.**
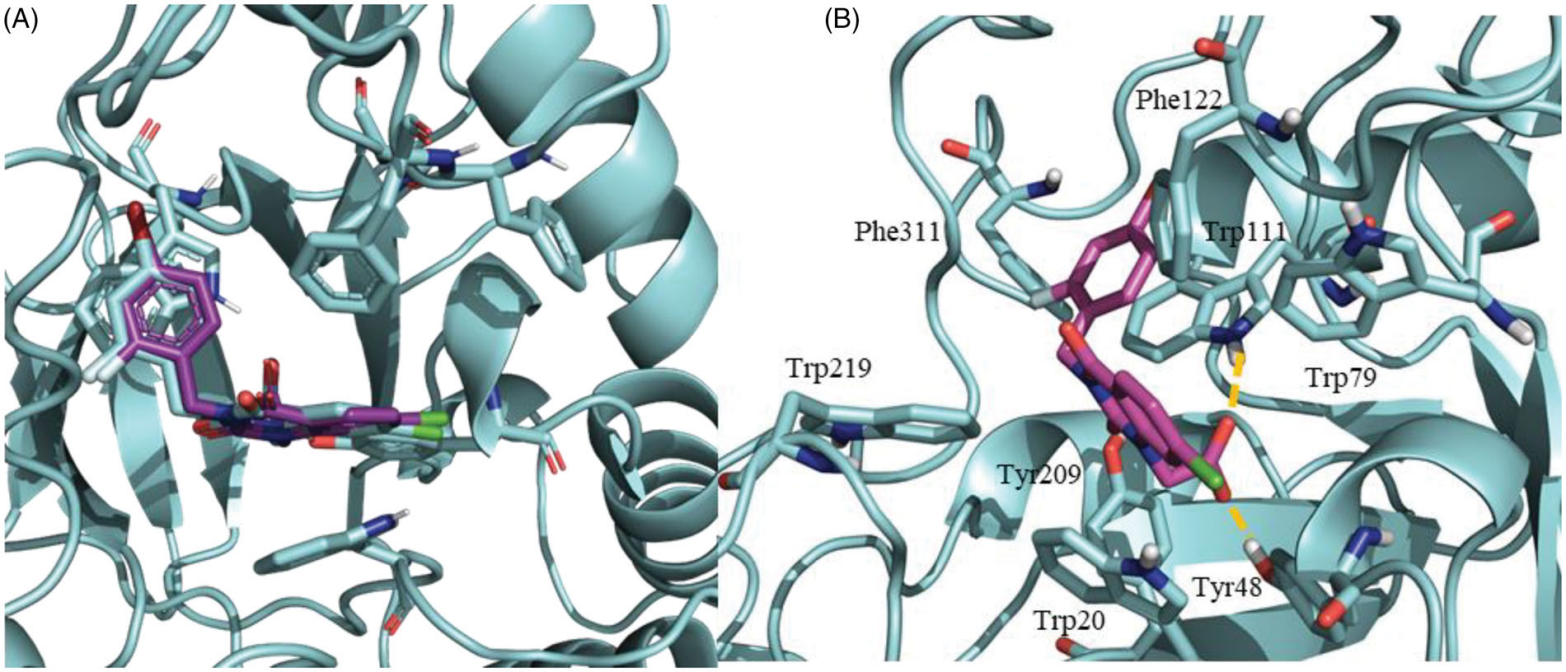
(A) Superimposition of zenarestat in the crystal structure 1IEI depicted in cyan and validation docking results depicted in purple show the high similarity between both poses in the human enzyme. (B) Detail of the zenarestat binding mode together with the main interactions found in the catalytic site of aldose reductase.

Docking studies were performed to assess the potential binding mode of NPD-1246 to *S. mansoni* aldose reductase. To validate our docking protocol, binding mode studies were first carried out with zenarestat, an inhibitor of the human aldose reductase crystallised with the enzyme (PDB code 1IEI)^45^. It was observed that the majority of conformations for zenarestat were grouped in a single cluster with very good binding energy profile around −9.75 kcal/mol. This cluster was visually analysed and the best pose was compared to the crystal structure already available. The docking binding pose of zenarestat (depicted in purple) is almost identical to the one that was previously crystallised (shown in cyan) (Figure 6(A)). In a similar way to the crystal structure, the main interactions found are with aromatic residues from the catalytic site: hydrogen bonds with Tyr48 and Trp111, and several important aromatic interactions mainly critical with Trp20 and Trp111 (Figure 6(B)).

**Figure 7.**
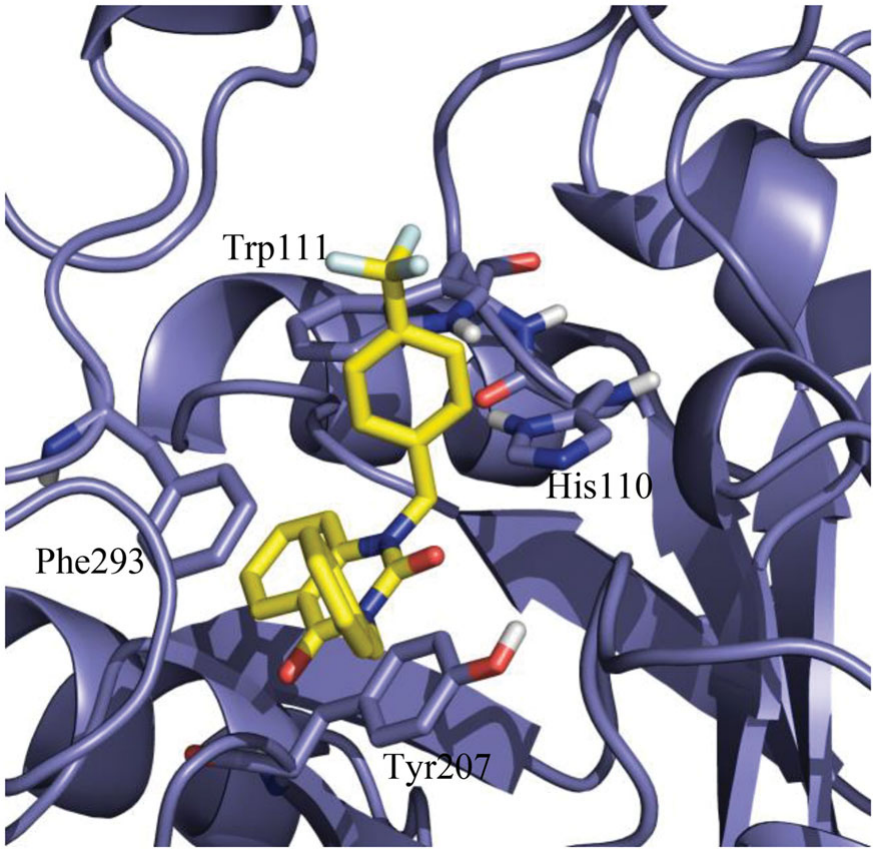
Detail of the binding mode of NPD-01246 in the *S. mansoni* aldose reductase binding site.

Once the protocol was validated, the potential binding mode of NPD-1246 in the aldose reductase of *S. mansoni* model was studied. Due to the fact that the model was based on 4HBK crystal, an apo structure of the *S. japonicum* aldose reductase, IFD studies^40^^,^^41^ were performed. Once the model was optimised, a final docking with NPD-1246 was carried out using the previously validated protocol. A clear defined cluster was obtained in which the vast majority of the docking poses were grouped with very similar conformations. The best-ranked pose of this cluster, with a binding energy around −9.5 kcal/mol, was selected as representative of the binding mode of NPD-1246 in the protein. As shown in Figure 7, the aromatic interactions are responsible for the stability of the ligand in the catalytic binding site, driving the ligand-binding process. Tyr207 and Phe293 are key to the stability, making face-to-face Π interactions with the quinazoline scaffold, while His110 and Trp111 are important making face-to-edge π interactions. As expected, due to the high similarity in terms of chemical structure between NPD-1246 and the novel derivatives, **9** and **10**, the main interactions with the enzyme are maintained (Supplementary Figure S5).

## Discussion

A previous phenotypic screening allowed us to successfully classify and identify promising compounds for further development based on antischistosomal *in vitro* potency. The selected quinazoline NPD-1246 showed an EC_50_ value of 50 µM in both males and females and a significant reduction in egg numbers at lower concentrations (Figure 1)^14^. Because metabolic stability is an important drug-like property to be considered for *in vivo* studies, the stability of NPD-1246 in the presence of mouse S9 microsomal fraction was checked. Unfortunately, the compound was extensively metabolised through phase-I metabolism (Table 1). Nevertheless, to check the *in vivo* activity potential, concomitant dosing with the CYP_450_ inhibitor ABT was carried out in the experimental *S. mansoni*-infected mouse model (control, NPD-1246, PZQ and combination of both).

NPD-1246 at 20 mg/kg orally showed a modest but significant reduction in total worm and intestinal egg loads together with an increase in the percentage of dead eggs. No significant differences were observed after administration at 10 mg/kg. More relevant to note is the synergy with PZQ when administered concomitantly with NPD-1246 at 10 mg/kg each (Figure 2). Reduction of total worms was 80% vs. 63% for PZQ alone with a complete disappearance of immature eggs and significantly increased percentages of dead eggs (84% vs. 66% for PZQ). This fact is of pivotal importance because eggs are responsible for the pathology and transmission of the disease^1^.

These results encouraged us to design NPD-1246 derivatives with improved metabolic stability. SMARTCyp^22^ software allowed us to identify position C6 in the quinazoline scaffold and *meta*- and *para*-positions of the benzyl substituents in the N1 as the most susceptible sites for metabolism by CYP_450_ (Figure 3). Taking these predictions into account, a series of chemically related compounds with these positions blocked were synthesised following a two-step synthetic procedure (Scheme 1). The new quinazolines **2**–**13** were tested *in vitro* against *S. mansoni* and their activity compared with the parent compound NPD-1246 (Tables 2–4). Two of them, **9** and **10** showed similar potency results as NPD-1246 but with an improved metabolic stability (Table 5), representing a significant improvement with respect to the original compounds.

The established antischistosomal potential of the quinazoline derivatives prompted us to investigate their MOA since target identification is becoming increasingly important to avoid/overcome drug resistance. The analysis of results obtained separately with three complementary computational methodologies (Supplementary Tables S1, S2 and Figure 4) led us to propose aldose reductase as the potential drug target for the quinazoline compounds.

*S. japonicum* aldose reductase was previously crystallised and antischistosomal activity of an inhibitor bearing two linked anthraquinone scaffolds was reported^46^. Although the role of *S. japonicum* aldose reductase is not fully understood, aldose reductase is believed to be an important antioxidant component in other organisms^48^^,^^49^. Moreover, antioxidant defense is an essential mechanism for schistosomes to face the damage from host immune and self-generated reactive oxygen species (ROS) and a number of redox-associated proteins have been already considered as key enzymes for drug development^50–53^. Based on these evidences, and after the cloning and characterisation of *S. japonicum* aldose reductase, it was proposed as a potential drug target for schistosomiasis due to its possible role in the worm antioxidant mechanism^54^.

All in all, these facts increased our interest in the quinazoline compounds as a new chemical class of inhibitors of this enzyme and a homology model of the *S. mansoni* aldose reductase was built based on the crystal structure of the *S. japonicum* counterpart^46^ for checking the binding mode of NPD-1246. The catalytic site of aldose reductase in *S. mansoni* is highly hydrophobic with a high number of aromatic residues such as Trp20, Tyr48, Trp79, Trp111, Phe122 or Tyr207 among others, highlighted in the sequence alignment (Supplementary Figure S3). Docking calculations indicated that NPD-1246 and new quinazolines **9** and **10** are able to bind the catalytic site of the enzyme through important interactions with aromatic residues, such as Tyr207, His110, Trp111 and Phe293 (Figure 7 and Supplementary Figure S5).

In conclusion, this study showed the antischistosomal potential of a new series of quinazoline derivatives through *in vitro* and *in vivo* studies and successful design of new derivatives to overcome the metabolic stability issues of the parent compound NPD-1246. The putative molecular target aldose reductase was identified by using complementary computational tools. This enzyme emerges as a new potential target to develop antischistosomal agents while the new quinazolines **9** and **10** represent improved candidates for further evaluation and development.

## Supplementary Material

Supplemental Material
