# Supplementary material for: Deciphering the enzymatic target of a new family of antischistosomal agents bearing a quinazoline scaffold using complementary computational tools
Source: J Enzyme Inhib Med Chem. 2020 Jan 15;35(1):511–23. doi: 10.1080/14756366.2020.1712595 (PMC7717570; doi:10.1080/14756366.2020.1712595)
Supplement: Supplemental Material [file IENZ_A_1712595_SM3058.pdf]

Supporting information for:

Deciphering the enzymatic target of a new family of antischistosomal agents bearing a quinazoline scaffold using complementary computational tools

Victor Sebastian-Perez,[a,§] Alfonso García-Rubia,[a,§] Sayed H. Seif el-Din,[b] Abdel-Nasser A. Sabra,[b] Naglaa M. El-Lakkany,[b] Samia William,[c] Tom L. Blundell,[d] Louis Maes,[e] Ana Martinez,[a] Nuria E. Campillo,[a] Sanaa S. Botros,[b,*] Carmen Gil[a,*]

Contents:

- Page S2: Figures S1-S2
- Page S3: Figure S3-S4
- Page S4: Figure S5
- Page S5: Table S1
- Page S6: Table S2
- Page S7: Table S3

[e]Centro de Investigaciones Biológicas (CIB-CSIC), Madrid, Spain

[a]Pharmacology Department, Theodor Bilharz Research Institute, Warrak El-Hadar, Imbaba, P.O. Box 30, Giza 12411, Egypt

[b]Parasitology Department, Theodor Bilharz Research Institute, Warrak El-Hadar, Imbaba, P.O. Box 30, Giza 12411, Egypt

[d]Department of Biochemistry, University of Cambridge, 80 Tennis Court Road, Cambridge, CB2 1GA, United Kingdom

[e]Laboratory for Microbiology, Parasitology and Hygiene (LMPH), University of Antwerp, Belgium

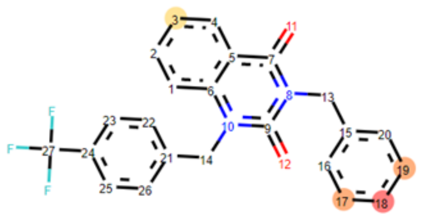

2C9 Ranking	Atom	2C9 Score	Energy	2DSASA	Span2end	COO-Dist	Similarity
1	C.18	90.8	86.3	36.2	0	7	1.0
2	C.17	102.6	86.3	34.3	1	6	1.0
3	C.3	114.5	86.3	32.7	3	6	1.0
4	C.16	114.6	86.3	29.4	2	5	0.7
5	C.23	114.8	86.3	24.5	3	6	0.7
6	C.13	118.7	66.4	21.6	4	3	0.4
7	C.14	118.7	66.4	20.8	6	3	0.4
8	C.2	120.8	80.8	32.7	4	5	1.0
9	C.4	121.1	80.8	26.1	4	5	1.0
10	C.22	126.6	86.3	25.0	4	5	0.7
11	C.1	132.5	86.3	25.7	5	4	0.7

Figure S1. Metabolic site prediction using SMARTCyp web server for NPD-1246. The atoms in the molecule are ranked according to the probability of being metabolized by the CYP_{2C9}.

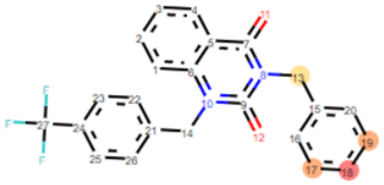

2D6 Ranking	Atom	2D6 Score	Energy	2DSASA	Span2end	N+dist	Similarity
1	C.18	84.9	86.3	36.2	0	0	1.0
2	C.17	91.6	86.3	34.3	1	0	1.0
3	C.13	92.4	66.4	21.6	4	0	0.4
4	C.14	92.4	66.4	20.8	6	0	0.4
5	C.16	98.5	86.3	29.4	2	0	0.7
6	C.3	105.1	86.3	32.7	3	0	1.0
7	C.23	105.4	86.3	24.5	3	0	0.7
8	C.2	106.3	80.8	32.7	4	0	1.0
9	C.4	106.6	80.8	26.1	4	0	1.0
10	C.1	112.1	86.3	25.7	5	0	0.7
11	C.22	112.1	86.3	25.0	4	0	0.7

Figure S2. Metabolic site prediction using SMARTCyp web server for NPD-1246. The atoms in the molecule are ranked according to the probability of being metabolized by the CYP_{2D6}.

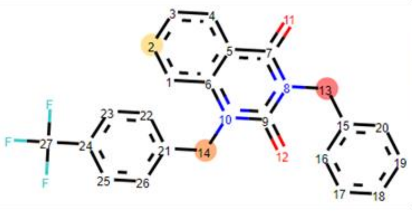

3A4 Ranking	Atom	3A4 Score	Energy	2DSASA	Span2end	Relative Span	Similarity
1	C.13	59.8	66.4	21.6	4	0.7	0.4
2	C.14	61.0	66.4	20.8	6	0.6	0.4
3	C.2	73.8	80.8	32.7	4	0.7	1.0
4	C.4	74.0	80.8	26.1	4	0.7	1.0
5	C.18	76.9	86.3	36.2	0	1.0	1.0
6	C.17	77.5	86.3	34.3	1	0.9	1.0
7	C.16	78.3	86.3	29.4	2	0.9	0.7
8	C.3	78.7	86.3	32.7	3	0.8	1.0
9	C.23	79.0	86.3	24.5	3	0.8	0.7
10	C.22	79.6	86.3	25.0	4	0.7	0.7
11	C.1	80.1	86.3	25.7	5	0.6	0.7

Figure S3. Metabolic site prediction using SMARTCyp web server for NPD-1246. The atoms in the molecule are ranked according to the probability of being metabolized by the CYP_{3A4}.

```
ALDR_HUMAN   MASRLLLNNGAKMPILGLGTWKSPPGQVTEAVKVAIDVGYRHIDCAHVYQNENEVGVAIQ 60
Q5DD64_SCHJA -MEPLKMNNGRSIPVIGLGTWNSPPGEVGAAVKKALEIGYRHLDCAYVYRNEAEIGEALE 59
G4LXS0_SCHMA -MESLKMNNGRSIPIIGLGTWNSPPGEVGVAVKKALEVGYRHLDCAYVYRNEAEIGGALE 59
               . * :*** .:*::*****:****:*  *** *:::****:***:**:** *:* *::

ALDR_HUMAN   EKLREQVVKREELFIVSKLWCTYHEKGLVKGACQKTLSDLKLDYLDLYLIHWPTGFKPGK 120
Q5DD64_SCHJA NALNSLRLKREDIFITSKLWNTFFRPEHVRKACEETLKNLRLNYLDLYLIHWPVPLKHGG 119
G4LXS0_SCHMA CSLKSLNLKREDVFVTSKLWNTFFRPEHVRKACEETLKNLRLKYLDLYLIHWPVPFQYGE 119
                *..  :***::*:.**** *:..    *: **::**.:*:*.**********. :: *

ALDR_HUMAN   EFFPLDESGNVVPSDTNILDTWAAMEELVDEGLVKAIGISNFNHLQVEMILNKPGLKYKP 180
Q5DD64_SCHJA DLFPTDSNGQLCLDNVPHEDTWKEMEKLVDEGLVKSIGLSNFNKRQIQNILEHC--RIKP 177
G4LXS0_SCHMA CLFPTDSNGNFCVDEVPHEETWKEMEKLVDDGLVKSIGLSNFNKRQIENILKHC--RIKP 177
              :** *..*:.  .:.   :**  **:***:****:**:****: *:: **::    : **

ALDR_HUMAN   AVNQIECHPYLTQEKLIQYCQSKGIVVTAYSPLGSPDRPWAKPEDPSLLEDPRIKAIAAK 240
Q5DD64_SCHJA ANLQIEIHANFPNIKLVEYAQSVGLTVTAYAPLGSPAH---SPGKVNLLTKPCVLEIAHR 234
G4LXS0_SCHMA ANLQIEIHANFPNIQLVEYAQSIGLTVTAYAPLGSPAA---SPGRVDLLMEPWVLQIAKH 234
             *  *** *  : : :*::*.** *:.****:*****     .*   .** .* :  ** :

ALDR_HUMAN   HNKTTAQVLIRFPMQRNLVVIPKSVTPERIAENFKVFDFELSSQDMTTLLSYNRNWRVCA 300
Q5DD64_SCHJA HKKTPAQVLLRYLLQRKLIVVPKSVTFKRIEENFQVFDFQLSNEEMHELNTESLNERQFT 294
G4LXS0_SCHMA HGKTPAQVLLRYLIQRNLIIVPKSVTPKRIEENFGVFDFQLSKEEMHELNTKGLNERQFK 294
             * ** ****:*: :**:*:::***** :** *** ****:**.::*  * : . * *

ALDR_HUMAN   LLSCTSHKDYPFHEEF 316
Q5DD64_SCHJA LLQMSGHQEYPFKEEY 310
G4LXS0_SCHMA LLKMANHSEYPFKDAY 310
             **. :.*.:***:: :
```

Figure S4. Multiple alignment of the different aldose reductase sequences in the human and in the *Schistosoma* parasite. Key aromatic residues from the catalytic site are highlighted.

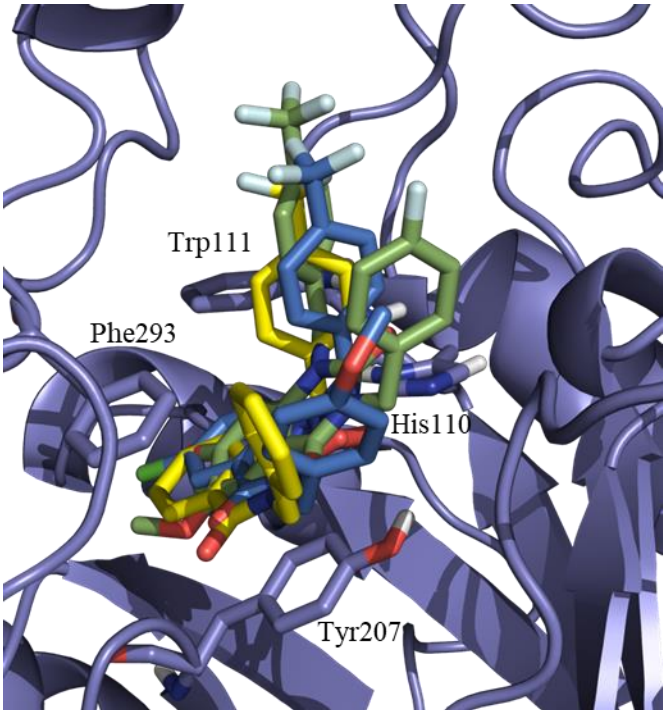

Figure S5. Detailed view of the predicted binding mode of compounds **9** (blue), **10** (green) in the *S. mansoni* aldose reductase binding site. A superposition with compound NPD-1246 (yellow) is displayed showing a high degree of similarity.

Table S1. Target ranking obtained according to the PPB results. A list of the 35 best ranking results is shown.

ChEMBL-ID	ChEMBL-Name	Name
1784	GLP1R	Glucagon-like_peptide_1_receptor
1293258	SMAD3	Mothers_against_decapentaplegic_homolog_3
2026	AMPC	Beta-lactamase_AmpC
1075094	NFE2L2	Nuclear_factor_erythroid_2-related_factor_2
1293224	MAPT	Microtubule-associated_protein_tau
2842	TARDB	TAR_DNA-binding_protein_43
2146309	MTOR	Serine/threonine-protein_kinase_mTOR
1741220	CLPP	ATP-dependent Clp_protease_proteolytic_subunit
614818	BAZ2B	Bromodomain_adjacent_to_zinc_finger_domain_protein_2B
2094267	PDE4D	Phosphodiesterase_4
1947	THRB	Thyroid_hormone_receptor_beta-1
2146310	VPR	Aberrant_vpr_protein
1293238	IMPA1	Inositol_monophosphatase_1
4096	TP53	Cellular_tumor_antigen_p53
1293254	FTL	Ferritin_light_chain
4179	MAPK9	c-Jun_N-terminal_kinase_2
2276	MAPK8	c-Jun_N-terminal_kinase_1
240	KCNH2	HERG
275	PDE4B	Phosphodiesterase_4B
4040	MAPK1	MAP_kinaseERK2
4377	GNAS	Guanine_nucleotide-binding protein_G(s),_subunit_alpha
6032	EHMT2	Histone-lysine_N methyltransferase,_H3_lysine-9_specific_3
1293278	GMNN	Geminin
1795085	ATXN2	Ataxin-2
5567	NA	Luciferin_4-monooxygenase
1293232	SMN1	Survival_motor_neuron_protein
1075051	DHFR	Dihydrofolate reductase
2104	P2RX4	P2X_purinoceptor_4
5409	GPBAR1	Gprotein_coupled bile_acid_receptor_1
3816	PLA2G4A	Cytosolic_phospholipase_A2
3081	AKR1B1	Aldose reductase
4361	MCL1	Induced_myeloid_leukemia_cell_differentiation_protein_Mcl-1
218	CNR1	Cannabinoid_CB1_receptor
1255150	GPBAR1	G-protein_coupled bile_acid_receptor_1
3563	NA	Cruzipain

Table S2. Target ranking obtained according to the SEA results. A list of the 35 best ranking results is shown.

Target Key	Target Name	Description	p-Value	MaxTC
PGES2_HUMAN+5	PTGES 2	Prostaglandin E synthase 2	1.346e-50	0.39
RORB_HUMAN+5	RORB	Nuclear receptor ROR-beta	1.758e-37	0.38
RGS4_HUMAN+5	RGS4	Regulator of G-protein signaling 4	2.607e-35	0.43
HPRK_BACSU+5	hprK	HPr kinase/phosphorylase	8.367e-33	0.33
RGS8_HUMAN+5	RGS8	Regulator of G-protein signalling 8	8.791e-31	0.35
FABI_ECOLI+5	fabl	Enoxyl-(acyl-carrier-protein) reductase (NADH) Fabl	7.338e-28	0.32
RORA_HUMAN+5	RORA	Nuclear receptor ROR-alpha	2.818e-27	0.38
DYR_BOVIN+5	DHFR	Dihydrofolate reductase	5.279e-26	0.33
FABP4_HUMAN+5	FABP4	Fatty acid-binding protein adipocyte	1.27e-24	0.44
KCNN2_RAT+5	Kcnn2	Small conductance calcium-activated potassium chappel protein 2	5.84e-23	0.31
ALD1_RAT+5	Akr1b7	Aldose reductase-related protein 1	3.998e-21	0.32
TLR7_HUMAN+5	TLR7	Toll-like receptor 7	4.768e-21	0.37
FABI_STAAR+5	fabl	Enoyl-(acyl-carrier-protein) reductase [NADPH] Fabl	6.077e-21	0.33
RAD1_HUMAN+5	RAD1	Cell cycle checkpoint protein RAD1	1.718e-17	0.34
AK1A1_HUMAN+5	AKR1A 1	Alcohol dehydrogenase [NADP(*)]	3.331e-16	0.32
ALDR_RAT+5	Akr1b1	Aldose reductase	1.665e-15	0.42
PA2GA_HUMAN+5	PLA2G2 A	Phospholipase A2, membrane associated	5.44e-15	0.38
PPARG_HUMAN+5	PPARG	Peroxisome proliferator-activated receptor gamma	1.05e-13	0.41
MALX3_YEAST+5	IMA1	Oligo-1,6-glucosidase IMA1	2.858e-13	0.31
FABPH_HUMAN+5	FABP3	Fatty acid-binding protein heart	3.2e-12	0.32
PSA_HUMAN+5	NPEPP S	Puromycin-sensitive aminopeptidase	5.592e-12	0.32
AK1BA_HUMAN+5	AKR1B 10	Aldo-keto reductase family 1 member B10	7.999e-12	0.44
ALDR_HUMAN+5	AKR1B 1	Aldose reductase	8.602e-12	0.34
FABP5_HUMAN+5	FABP5	Fatty acid-binding protein epidermal	9.502e-12	0.31
P2RX4_HUMAN+5	P2RX4	P2X purinoceptor 4	1.041e-11	0.51
CAC1B_RAT+5	Cacna1 b	Voltage-dependent N-type calcium channel subunit alpha-1B	4.116e-11	0.35
BGAL_ECOLX+5	lacZ	Beta-galactosidase	2.645e-10	0.29
PKR1_HUMAN+5	PROKR 1	Prokineticin receptor 1	3.503e-10	0.30

SETD7_HUMAN+5	SETD7	Histone-lysine N-methyltransferase SETD7	5.753e-10	0.30
MDHM_HUMAN+5	MDH2	Malate dehydrogenase mitochondrial	6.52e-10	0.36
PD2R2_HUMAN+5	PTGDR 2	Prostaglandin D2 receptor 2	7.745e-10	0.43
AA3R_HUMAN+5	ADORA 3	Adenosine receptor A3	2.644e-09	0.40
EGLN1_HUMAN+5	EGLN1	Egl nine homolog 1	3.855e-09	0.39
PDE8A_HUMAN+5	PDE8A	High affinity cAMP-specific and IBMX-insensitive 3´,5´-cyclic phosphodiesterase 8A	5.204e-09	0.29

Table S3. Validation of the *S. mansoni* model using as a template the aldolase reductase from *S. japonicum* (PDE code 4HBK) considering geometrical and energetical parameters.

QMEAN4	Ramachandran			Verify3D	Errat
	Favoured	Allowed	Outlier		
-0.23	302 (98.1%)	5 (1.6%)	1 (0.3%)	92.9%	93.3%
